# Glucokinase Gene Mutations: Structural and Genotype-Phenotype Analyses in MODY Children from South Italy

**DOI:** 10.1371/journal.pone.0001870

**Published:** 2008-04-02

**Authors:** Nadia Tinto, Adriana Zagari, Marina Capuano, Alfonso De Simone, Valentina Capobianco, Gerardo Daniele, Michela Giugliano, Raffaella Spadaro, Adriana Franzese, Lucia Sacchetti

**Affiliations:** 1 Dipartimento di Biochimica e Biotecnologie Mediche, Università di Napoli “Federico II” and CEINGE Biotecnologie Avanzate, Napoli, Italia; 2 Dipartimento delle Scienze Biologiche, Università di Napoli “Federico II” and CEINGE Biotecnologie Avanzate, Napoli, Italia; 3 Dipartimento di Pediatria, Università di Napoli “Federico II”, Napoli, Italia; Ecole Normale Supérieure de Lyon, France

## Abstract

**Background:**

Maturity onset diabetes of the young type 2 (or GCK MODY) is a genetic form of diabetes mellitus provoked by mutations in the glucokinase gene (*GCK*).

**Methodology/Principal Findings:**

We screened the *GCK* gene by direct sequencing in 30 patients from South Italy with suspected MODY. The mutation-induced structural alterations in the protein were analyzed by molecular modeling. The patients' biochemical, clinical and anamnestic data were obtained. Mutations were detected in 16/30 patients (53%); 9 of the 12 mutations identified were novel (p.Glu70Asp, p.Phe123Leu, p.Asp132Asn, p.His137Asp, p.Gly162Asp, p.Thr168Ala, p.Arg392Ser, p.Glu290X, p.Gln106_Met107delinsLeu) and are in regions involved in structural rearrangements required for catalysis. The prevalence of mutation sites was higher in the small domain (7/12: ∼59%) than in the large (4/12: 33%) domain or in the connection (1/12: 8%) region of the protein. Mild diabetic phenotypes were detected in almost all patients [mean (SD) OGTT = 7.8 mMol/L (1.8)] and mean triglyceride levels were lower in mutated than in unmutated GCK patients (p = 0.04).

**Conclusions:**

The prevalence of GCK MODY is high in southern Italy, and the GCK small domain is a hot spot for MODY mutations. Both the severity of the *GCK* mutation and the genetic background seem to play a relevant role in the GCK MODY phenotype. Indeed, a partial genotype-phenotype correlation was identified in related patients (3 pairs of siblings) but not in two unrelated children bearing the same mutation. Thus, the molecular approach allows the physician to confirm the diagnosis and to predict severity of the mutation.

## Introduction

Maturity onset diabetes of the young (MODY; MIM #606391) is a genetically and clinically heterogeneous form of diabetes mellitus, characterized by an early age at onset, a primary defect in beta-cell function and an autosomal dominant inheritance [1]. Among the different types of MODY diabetes described thus far, each of which is due to a different gene mutation (HNF4A, GCK, HNF1A, IPF1, HNF1B, NEUROD1, CEL) [2], [3] the GCK MODY form is provoked by mutations in the glucokinase gene (*GCK*; MIM#138079).

The glucokinase gene on chromosome 7p15.3-p15.1 consists of 12 exons that span ∼45.169 bp and encode a 465-amino-acid protein [4], and three tissue-specific isoforms are known [5]. Thus far, about 200 GCK mutations have been reported and its frequency is higher in European Caucasians, particularly in those from France and Italy [6]. The identification of a GCK mutation in subjects whose clinical phenotype is suggestive of MODY usually distinguishes patients with a benign prognosis (GCK MODY) from those with a severe hyperglycemia (HNF1A MODY and other MODY forms) because the diagnosis cannot be always made on clinical grounds alone.

Glucokinase (also called hexokinase IV) catalyzes the ATP-dependent phosphorylation of glucose to glucose-6-phosphate. It is homologous to hexokinases I, II and III, but its lower affinity for glucose, restricted localization to a few cell types and peculiar kinetic properties, compared to those of the other hexokinases, confer it distinctive properties. Indeed, GCK acts as a glucose sensor in the pancreas and liver, and presents a peculiar sigmoidal glucose saturation curve, which indicates cooperative behaviour.

Elucidation of crystal structure of GCK yielded data that could help to establish structure-function correlations [7]. Indeed, the protein folds into two domains known as the small and the large domain with the glucose binding site in between. An unexpected “super-open” form and a “closed” form of the enzyme were identified [7]; the latter is similar to the form found in the hexokinase I structure [8]. The two forms differ in the relative spatial orientation of the two domains. The catalytic mechanism requires dramatic GCK conformational changes. In fact, when bound to glucose, the enzyme goes from the super-open inactive form to the closed form. Therefore, the inter-domain motions are crucial for the enzyme activity.

In this study, we report the identification of 9 novel and 3 known mutations of the *GCK* gene in children from south Italy. All mutations co-segregated with the diabetic phenotype in the respective families and resulted in perturbation of the 3D structure of the protein. Our data show that molecular screening is useful in the diagnosis of MODY because it allows to confirm the diagnosis and to predict severity of the mutation.

## Methods

### Subjects

Thirty patients aged 1–14 years, 9 boys and 21 girls whose clinical presentation was suggestive of MODY were selected for *GCK* gene screening from among 240 diabetic children seen in the Paediatric Clinic of our Medical School between 2001 and 2006. Other MODY genes were not investigated. Most patients were unrelated; 6 were except (3 pairs of siblings, 1: M022–M023, 2: M024–M025, 3: M028–M029). Inclusion criteria were: early onset (<25 years) of diabetes, mild hyperglycemia, no autoimmune markers of type I diabetes, without obesity [c.o. body mass index (BMI) *z* score >2] and family history of diabetes for at least two consecutive generations [1], [9]. No treatment was administered to the patients and no diabetes complications were evident up to diagnosis. A fasting blood sample was drawn from both parents of mutated patients and from 100 unrelated controls, who came from the same geographical area, and used for the GCK molecular characterization. The parents of all subjects gave their written informed consent to the study. The research was conducted according to Helsinki II declaration and approved by the ethics committee of our Faculty.

### Clinical and anamnestic examination

We collected the following data for each patient upon diagnosis: age, birth weight, family history of diabetes and/or other diseases and BMI. The BMI was transformed into BMI-z-score (z-BMI) based on the Centre for Disease Control normative curves [10], [11].

### Biochemical analyses

The following biochemical parameters were measured on fasting blood samples: plasma glucose (FPG) by the enzymatic hexokinase method and triglycerides by the standard enzymatic method (Hitachi Modular, Tokyo, Japan); glycosylated haemoglobin (HbA1c) by HPLC (HLC-723 G7 TOSOH Bioscience Tokyo, Japan); serum insulin by the chemiluminescence method (Immulight 2000; Medical System Genoa, Italy). After an oral glucose dose of 0.75g/kg body weight (maximum 75 g), oral glucose tolerance test (OGTT) was evaluated on blood samples collected every 30 min up to 2 h. The first-phase insulin response (FPIR) was calculated as the sum of T+1 and +3 min serum insulin concentrations, evaluated after an i.v. glucose dose of 0.5 g/kg body weight in 3 min [12].

### DNA extraction

Genomic DNA from patients, parents and controls was extracted from a blood sample plus EDTA using Nucleon BACC 2 kit (Amersham Biosciences Europe, Milan, Italy).

### Sequence analysis

Exons and flanking intron regions of *GCK*, including tissue specific variants of exon 1, were amplified by PCR using previously reported primers (exons 1a, 2, 3, 4) [13] or chosen by the Primer 3 program (exons 1b, 1c, 5, 6, 7, 8, 9 and 10) [14]. The PCR mixture contained in a final volume of 50 µl: 20 µM each primer, 1x PCR buffer (Applied Biosystems, Foster City, CA, USA), 10 µM each deoxynucleotide triphosphate, 2.5 U of *Taq* DNA polymerase (Applied Biosystems) and 200 ng of genomic DNA. Each PCR was performed on Gene-Amp PCR system 9700 thermocycler (Applied Biosystems) and consisted of a initial denaturation step at 95°C for 5 min and a final extension at 72°C for 7min.

The primers and PCR conditions are detailed in table 1. Product sizes were evaluated by agarose gel electrophoresis and amplicons were sequenced in both directions using the Big Dye terminator sequencing kit (Applied Biosystems) on ABI PRISM sequencing apparatus 3730 (Applied Biosystems). We preliminarily analyzed the *GCK* coding sequence of a healthy subject by sequence analysis and verified that it overlapped the wild-type reference sequence (GenBank NM_000162). All sequences of the patients were analyzed and compared with the wild-type published reference sequence with the ABI Seqscape software v2.5 (Applied Biosystems).

**Table 1:** Primers used for PCR amplification of GCK exons and PCR conditions.

Exons^1^	Forward primer	Reverse primer	PCR annealing temperatures	PCR annealing times	PCR extension times (72°C)	Number of cycles
1a (2)	5′-TCCACTTCAGAAGCCTACTG	5′-TCAGATTCTGAGGCTCAAAC	60°C	30”	60”	35
1b	5′-AGCAGGCAGGAGCATCTCTG	5′-GCTGCTCTCCCAGTGCAAAG	62°C	20”	60”	30
1c	5′-GGCCAACTGCTACTTGGAAC	5′-AGGAGGTGAGAAGCCTGGAG	54°C	20”	30”	35
2 (2)	5′-TGCAGATGCCTGGTGACAGC	5′-CACAGCTGCTTCTGGATGAG	60°C	15”	30”	30
3 (2)	5′-TAATATCCGGCTCAGTCACC	5′-CTGAGATCCTGCATGCCTTG	57°C	15”	30”	30
4 (2)	5′-TAGCTTGGCTTGAGGCCGTG	5′-TGAAGGCAGAGTTCCTCTGG	62°C	20”	60”	30
5/6	5′-TCTGAGCCTGTTTCCTCAGC	5′-GGCCCTTGAAGCCTGTTGTA	57°C	20”	45”	35
7	5′-CCAGACAAAGCAGAGACAGG	5′-TGCTTTTCCCCAGAGTTGTT	54°C	20”	45”	35
8	5′-TGGCTCATTAACGAGGGAAA	5′-CTGAGACCAAGTCTGCAGTG	51°C	15”	45”	35
9	5′-CCCTCCCTGGAGAACGAGAG	5′-AATCTTGGAGCTTGGGAACC	60°C	20”	45”	35
10	5′-GAGTCTTCTCGACCCCCTTG	5′-CACCGAAAAACTGAGGGAAG	55°C	20”	45”	35

1 GenBank: accession n° (AH005826)

2 Taken from Stoffel M. et al. 1992 Proc Natl Acad Sci USA 89:7698–7702.

All mutations were also validated on a second PCR product. Finally, the children's parents and 100 unrelated healthy individuals were screened for these mutations.

Conservation of residues was evaluated from a multiple sequence alignment of 15 sequences in the PFAM349 and PFAM3724 protein families [15]. The result was validated by a multiple sequence alignment of 341 protein sequences homolog to GCK and extracted from the non redundant sequence database RefSeq, available online [16]. Furthermore, for the 1v4s structure, a multiple sequence alignment of 143 sequences in the ConSurf_HSSP database [17] was carried out. The outcomes from the three approaches were in accordance.

### Statistical analysis

Continuous variables are reported as mean (SD) and comparisons among variables were made with the *t- test.* Inter-group differences were considered statistically significant at p<0.05.

We calculated the distribution in the large domain, in the small domain and in the connection region of the protein of our GCK mutations and of those described in literature.

### Mutations nomenclature

All mutations are described according to the recommended nomenclature available online [18]–[20]. Nucleotide numbers are derived from cDNA *GCK* sequence (GenBank NM_000162) considering nucleotide +1 the A of the first ATG translation initiation codon in the reference sequence.

### Molecular modelling

We used molecular modelling to investigate alterations of the GCK 3D structure and dynamics associated to the various mutations. The active form of GCK served as template because it has the highest resolution structure (2.3 Å, PDB code: 1v4s) [7]. Under the assumption that point mutations are likely to preserve the overall fold of the proteins, it follows the procedure for generating models. The first step of this procedure is the prompt substitution of the residues, which we did with the program MODELLER [21]. The program replaces the side chains by selecting the most abundant conformers and performs a simulated annealing procedure to optimize side chain conformations. This was followed by energy minimization in explicit solvent with the GROMACS program [22] using the GROMOS96 force field [23]. Finally, the system was optimized by means of a short molecular dynamics calculation of 1ns. The calculation was made by restraining the main chain conformations and allowing the side chain dynamics. The notation used for secondary structure was taken from Kamata et al. [7]. Accordingly, sequences 1–64 and 206–439 belong to the large domain, sequences 72–201 and 445–465 belong to the small domain, and sequences 65–71, 202–205 and 440–444 belong to the three loops connecting the domains.

## Results

**Figure 1:**
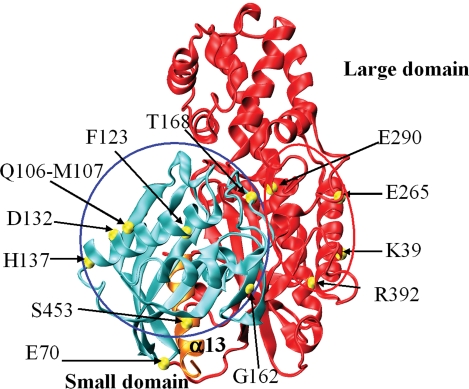
Distribution of the GCK mutations. The structure of GCK in the closed form (PDB code: 1v4s) is shown as cyan and red ribbons that represent the small and large domain, respectively. Orange ribbons show the α13 helix. Yellow spheres are the mutation sites. Red and blue circles indicate clusters of mutations in the large and small domain, respectively.

**Figure 2:**
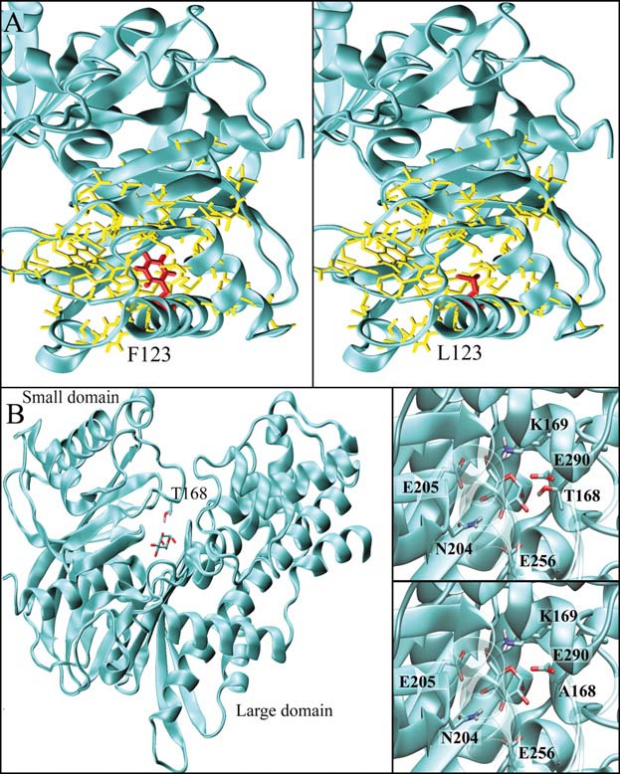
Structural features of mutations p.Phe123Leu and p.Asp168Ala GCK. The structure of GCK in the closed form (PDB code: 1v4s) is represented by cyan ribbons. A) Mutation p.Phe123Leu. Phe123 is structured inside the hydrophobic core of the small domain. Yellow sticks represent hydrophobic residues that constitute the core. Phe123 and Leu123 are represented by red sticks (left and right panel, respectively). B) The sticks represent residue Thr168 and glucose. The right panels show a close-up view of the glucose-binding cleft for the wild-type (top) and for the Ala168 mutant (bottom).

**Figure 3:**
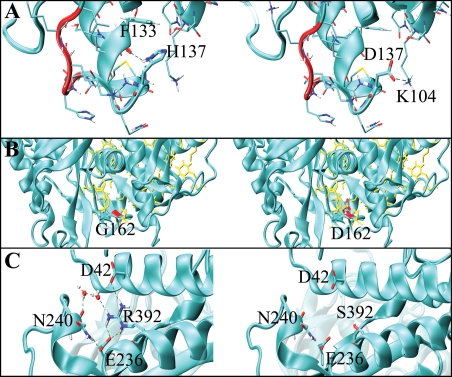
Structural features of mutations p.His137Asp, p.Arg392Ser and p.Gly162Asp GCK. The structure of GCK in the closed form (PDB code:1v4s) is shown as cyan ribbons. A) p.His137Asp. Loop 141–144, which is involved in GKRP binding, is in red. His137 is at the end of the α3 helix. His137 is a capping residue of the helix, which is terminated by the interaction between side chain of His137 and Phe133. Asp137 (right) is not able to replace His137 interactions but adds a new interaction with Lys104. B) p.Gly162Asp. Yellow sticks represent hydrophobic residues that constitute the core. The location of Gly162 is marked in red (left of the panel). Asp162 is on the right of the panel. C) p.Arg392Ser. Residues Asp42, Glu236, Asn240 and Arg392 are represented by yellow sticks. H-bonds are shown in green. The wild-type enzyme and the p.Arg392Ser mutant are on the left and right of the panel, respectively.

Table 2 shows the molecular characterization of the 16/30 our diabetic children who had mutations in the *GCK* gene. Ten patients were unrelated (two of these had the same mutation) and 6 were siblings from 3 families. Therefore, GCK MODY diagnosis was confirmed in these 16 patients. All mutations were detected at the heterozygous state, 9 mutations were novel; 8 of them caused variations in amino acid residues well conserved among species, and one produced a truncated protein of 289 amino acids (p.Glu70Asp, p.Phe123Leu, p.Asp132Asn, p.His137Asp, p.Gly162Asp, p.Thr168Ala, p.Arg392Ser, p.Glu290X, p.Gln106_Met107delinsLeu). Three other GCK mutations were already known; 2 of them caused truncated proteins (p.Lys39fsX6 and p.Ser453X) [24], [25] and 1 was a missense mutation (p.Glu265Lys) (Bellané-Chantelot C et al. Abstract, Diabetologia 1998; 41:A109, 423). The structural analysis of mutation sites indicated 3D clustering. In fact, among the GCK variants in our population, 7/12 (∼59%), 4/12 (33%) and 1/12 (8%) were localized in the small domain, in the large domain and in the connecting loops, respectively (Fig. 1). The effects of GCK mutations on the enzyme's 3D structure are reported in Table 2. Figures 2 and 3 show the structural features of some of the new mutations detected in our GCK MODY patients.

**Table 2:** GCK mutations in children from south Italy affected by GCK MODY

Patient code	Exons^a^	cDNA mutation^b^	AMINOACID CHANGE^C^	Domain localization /Secondary structure	Effect on protein 3D-structure	References
M001	8	c.868G>T	p.Glu290X Stop codon, truncated protein	Large domain/α7 helix	Truncated protein	Present study
M006						
M002	2	c.115delA	p.Lys39fsX6 Frameshift and stop codon	Large domain/α2 helix	Truncated protein	Prisco et al 2000
M003	9	c.1174C>A	p.Arg392Ser Positively charged Arg → polar Ser	Large domain/α11 helix	Disruption of H-bond and salt-bridge network	Present study
M005	10	c.1358C>A	p.Ser453X Stop codon	Small domain/α13 helix	Truncated protein Loss of C-term segment of α13 helix	Massa et al 2001
M009	4	c.409C>G	p.His137Asp Polar His → negatively charged Asp	Small domain/Last residue of α13 helix	Loss of α13 helix capping Variation of local interactions	Present study
M013	5	c.485G>A	p.Gly162Asp Apolar Gly → negatively charged Asp	Small domain/β strand 7	Introduction of a negative charge in a hydrophobic environment	Present study
M017	4	c.369C>G	p.Phe123Leu Aromatic hydrophobic Phe → hydrophobic Leu	Small domain/α3 helix	Reorganization dictated by an introduction of small cavity into a hydrophobic environment	Present study
M018	3	c.317_319delAGA	p.Gln106_Met107delinsLeu	Small domain/Edge β strand 4	Perturbation of the β-sheet	Present study
M019	2	c.210A>C	p.Glu70Asp Negatively charged Glu → negatively charged Asp	Connection/Loop spatially near α13 helix	Weakness of salt-link interaction with K458 (α13 helix)	Present study
M022^d^	5	c.502A>G	p.Thr168Ala polar Thr →nonpolar Ala	Small domain/Loop	Loss of H-bond between T168 and glucose	Present study
M023^d^						
M024^e^	7	c.793G>A	p.Glu265Lys Negatively charged Glu → positively charged Lys	Large domain/Loop	E265 is between R36 and R43. positively-charged K, provokes a dramatic rearrangement	Bellane-Chantelot C et al 1998
M025^e^						
M028^f^	4	c.394G>A	p.Asp132Asn Negatively charged Asp →polar Asn	Small domain/α3 helix	Mild structural alterations	Present study
M029^f^						

a GenBank: accession n° (AH005826)

b The reference cDNA sequence was obtained from GenBank (NM_000162) and +1corresponds to the A of the ATG translation initiation codon

c Swissprot accession n° P35557

d, e, f Sibling pairs

Table 3 shows the phenotypic characteristics of our mutated children at diagnosis. All mutations were also present in either the mother or the father of the GCK MODY patients in association with diabetes and mean birth weight did not differ between children who inherited GCK mutations from their father or mother (2.84 kg vs 2.98 kg). Almost but one patient had mild hyperglycemia [mean (range) FPG = 6.60 (5.7–7.9) mmol/L, M013 = 10.0 mmol /L], mildly altered OGTT [mean (SD) 2 h OGTT = 7.8 (1.8) mmol/L] and 2h increment always <2.8 except in two patients: M024 = 3.3 and M005 = 4.9; FPIR values between 24.6 and 156.0 µU/mL and slightly elevated levels of HbA1c [mean (SD) HbA1c = 6.1 (0.4) %]. The mean triglyceride concentration (0.58 mmol/L) was lower in our mutated patients (p = 0.04) than in unmutated GCK MODY patients (0.80 mmol/L) and below 50^th^ percentile of our laboratory age-related reference range (0.36–1.10 mmol/L).

**Table 3:** Biochemical and phenotypic characteristics of the children affected by GCK MODY

PATIENT CODE	SEX	BIRTH WEIGHT (kg)	AGE AT DIAGNOSIS (years)	BMI^a^ z-scores	FPG^b^ (mmol/L)	120 min OGTT^c^ (mmol/L)	FPIR^d^ (µU/ml)	HbA1c^e^ (%)	TRIGLYCERIDES (mmol/L)	AFFECTED FAMILY MEMBER
M001	F	1.80	10	−3.59	IFG	NGT	28.4	6.1	0.65	F
M002	F	2.70	1	----f	IFG	n.a.	33.4	6.4	2.26^g^	M
M003	F	3.30	8	0.67	IFG	NGT	112.7	6.7	0.99	M
M005	F	2.95	9	0.26	IFG	DM	88.0	6.5	0.45	F
M006	M	2.05	6	0.72	IFG	IGT	69.1	5.9	0.36	M
M009	M	3.25	6	1.56	DM	NGT	39.4	5.6	0.80	F
M013	M	2.97	7	−1.80	DM	IGT	24.6	5.9	0.64	M
M017	M	3.50	6	−0.59	IFG	NGT	n.a.	n.a.	0.57	M
M018	F	2.80	14	−0.60	IFG	NGT	130.5	6.5	0.49	F
M019	F	3.55	10	1.46	IFG	IGT	140.0	5.9	0.54	M
M022	F	2.95	8	1.60	IFG	NGT	n.a.	6.2	0.66	F
M023	M	3.40	9	0.67	IFG	NGT	156.0	6.4	0.82	F
M024	F	2.75	12	1.29	IFG	DM	104.0	6.5	0.40	F
M025	F	2.50	5	1.56	IFG	IGT	35.5	6.2	0.29	F
M028	F	2.85	12	1.42	IFG	NGT	n.a.	5.4	0.52	M
M029	M	2.91	7	0.74	IFG	NGT	n.a.	5.5	0.56	M

a BMI*z* scores: (calculated in children aged 2–18 years), see Materials and methods

b FPG: Fasting plasma glucose; (impaired fasting glucose [IFG] = 5.6–6.9 mmol/L; diabetes mellitus [DM] = ≥7.0 mmol/L)

c OGTT: Oral glucose tolerance test (normal glucose tolerance [NGT] <7.8 mmol/L; impaired glucose tolerance [IGT] = 7.8–11.0 mmol/L; DM = ≥11.1 mmol/L)

d FPIR: First phase insulin response (reference value: ≥60.0 µU/ml)

e HbA1c: glycosylated haemoglobin (reference value: 4.3–5.9%)

f Not available because patient M002 was <2 years old

g Not fasting nursling

n.a. Not available

## Discussion

In our Italian population, *GCK* gene mutations account for 6% (16/240) diabetic children and for 53% (16/30) with suspected MODY. This GCK MODY prevalence is in line with those detected in southern European countries, particularly in France and in Italy, namely between 8% and 56% [25]–[27]. An analysis of the 3D structure of the protein mutants yielded evidence of structural perturbations, which supports the GCK MODY-causing nature of these mutations. In particular, the p.Glu70Asp mutation (detected in 1 patient) substitutes the highly conserved glutamate with aspartate. These amino acids are polar acidic residues but the binding to lysine 458, in the α13 helix, appears to be weakened compared with the wild-type enzyme. This substitution could slightly modify enzymatic activity and/or stability. Substitution of glutamate by lysine is reported, in association with diabetes, to reduce the enzyme's glucose affinity but protein stability is preserved [28].

The p.Gln106_Met107delinsLeu mutation (detected in 1 patient) altered respectively, a poorly and moderately conserved amino acid. It produces a protein in which residues Gln106 and Met107 are substituted by a leucine. This mutation occurs on an edge strand of the β-sheet of the small domain. The β-sheet encompasses the α13 helix in the closed form. Because the conformational features of this region are essential for super-open/closed conversion, the deletion could influence GCK function. A gene deletion encompassing GCK exon 2 (p.Val16_Glu70del) has recently been described in a UK diabetic patient by multiplex ligation-dependent probe amplification assay and it co-segregated with early onset diabetes within the pedigree [29]. The p.Phe123Leu mutation (detected in 1 patient) altered a highly conserved amino acid. Phe 123 is located on the α3 helix within the small domain. Phe 123 is projected into the hydrophobic core of the domain thereby contributing to its stability. The substitution of a phenylalanine with a leucine is not dramatic in terms of hydrophobicity, however, it introduces a cavity in the hydrophobic core. This may affect inner surface complementarity thereby influencing the structural stability and the dynamical behaviour of the domain with consequences at a functional level.

In mutant p.Asp132Asn *(*detected in 2 related patients), the acidic negatively charged aspartate, located in the α3 helix that belongs to the small domain, is changed into the uncharged asparagine. Aspartate 132 is a poorly conserved amino acid and this variant probably provokes only mild structural alterations. In fact, the 2 siblings bearing this mutation had normal glucose tolerance. The p.His137Asp mutation (detected in 1 patient) altered a moderately conserved amino acid. The activity of hepatic GCK is regulated by the glucokinase regulatory protein (GKRP). This would act as an allosteric inhibitor of GCK that specifically binds to the super-open form. Indeed, mutational analyses [30] have shown that two GCK fragments, 51–52 and 141–144, are involved in such interactions. Sequence 141–144 follows the α3 helix that is terminated by His 137. Histidine, by interacting with the carbonyl of Phe133, is involved in helix capping. Mutation p.His137Asp introduces a negative charge in the region and, in our simulations, Asp137 does not exert a capping function, but strongly interacts with Lys104 by making a salt bridge. Accordingly, p.His137Asp may affect the conformational properties of fragment 141–144 thereby indirectly influencing the binding with GKRP. The p.His137Arg mutation has been described in association with diabetes [31].

The p.Gly162Asp mutation *(*detected in 1 patient) altered a highly conserved amino acid. Gly162 is located on the β-sheet that encloses the small domain hydrophobic core. p.Gly162Asp is one of the most dramatic mutations we identified because it introduces a negative residue inside the hydrophobic core. p.Gly162Asp very probably influences the stability of the core thereby altering the structure and dynamics of the domain. This scenario is indicative of functional impairment of the enzyme.

The p.Thr168Ala mutation *(*detected in 2 related patients) affected a conserved amino acid. The glucose-binding cleft is located at the interface between small and large domains. It is constituted by residues Glu256 and Glu290 from the large domain, Thr168 and Lys169 from the small domain, and Asn204 and Asp205 from the interconnecting region. Binding a glucose molecule requires a precise pattern of H-bonds between the substrate and GCK. Thr168 binds glucose, therefore the p.Thr168Ala substitution prevents the formation of the H-bond and probably perturbs the enzyme's binding affinity and efficiency. Mutation p.Thr168Ala has been described in patients affected by diabetes [32]; it greatly increased Vmax and resulted in a complete loss of cooperative behaviour associated with glucose binding, the 2 siblings bearing this mutation had normal glucose tolerance and impaired glycosylated hemoglobin. Glutamate 290 is a highly conserved residue involved in glucose binding. The p.Glu290X mutation *(*detected in 2 unrelated patients) introduces a stop codon and generates a truncated protein of only 289 amino acids, which is thus unable to function.

The p.Arg392Ser mutation (detected in 1 patient) alters a conserved amino acid. Arg392, is located on the α11 helix in the large domain and is involved in a local H-bond/salt bridge network. Arg392 is positively charged and makes a salt bridge with the negative residues Asp42 (α2) and Glu236. The H-bond network extends to two water molecules and residue Asn240. These residues, which are far in sequence, are relevant for the tertiary structure of the domain, in fact serine is unable to replace the wild-type Arg392 interactions. The p.Arg392Cys mutation was reported in co-segregation with hyperglycemia in pregnancy [33].

Three patients carried already known GCK mutations: p.Lys39fsX6, p.Ser453X and p.Glu265Lys. All these mutations were described in association with hyperglycemia. In particular, the Ser → Leu mutation at residue 453 was recently found to reduce GCK activity in a GCK MODY patient [34]. In our GCK MODY patients, the distribution of mutation sites in the GCK protein (59%, 33% and 8% in the small domain, large domain and in the connection region, respectively) differed from the distribution observed in European Caucasians and in other ethnic groups (41%, 58% and 1% in the small domain, large domain and in the connection region, respectively) [5], [35]. Consequently, the GCK small domain may be a hot spot for MODY mutations typical of Southern Italy. Interestingly, almost all the mutation sites we describe are in regions involved in structural rearrangements required for catalysis. This finding supports the notion that mutations may affect GCK function, which is intimately related to intermotion domain [7]. Our data confirm the association between low triglyceride values and GCK mutations and support a low rate of cardiovascular complications in GCK MODY diabetes [36]. Interestingly, the two patients (M001 and M013) with the lowest BMI *z* scores also had the lowest FPIR values, which is in line with the finding that, at low levels, insulin does not exert an anabolic effect [37].

Massa et al. [25] did not find an association between phenotype and genotype in GCK MODY patients. Two of our unrelated patients, M001 and M006, who both carried the p.Glu290X mutation, had a low birth weight but a different diabetic phenotype as evaluated by OGTT, FPIR tests and triglyceride level. In contrast, among the three pairs of siblings, each with the same mutation, two pairs (M022-M023, M028-M029) had almost identical metabolic phenotypes. In the third pair of siblings (M024-M025, aged 12 and 5 years respectively), the elder child was diabetic and the younger had impaired glucose tolerance. In these two patients, the p.Glu265Lys mutation provoked a dramatic rearrangement of GCK, which indicates a more severe prognosis. Thus, both the severity of the GCK mutation and the genetic background seem to play a relevant role in the GCK MODY phenotype.

In conclusion, all mutations detected in our diabetic children from south Italy co-segregated with the diabetic status in one or two family members of each patient and were not detected during the screening of 200 normal chromosomes. The new deletion and missense mutations produced an amino acid substitution at positions that are well conserved among several species. Finally, our data show that molecular screening is useful in the diagnosis of MODY because it allows one to confirm the diagnosis and to predict the prognosis as well as the clinical course of the patient.

## References

[1] Fajans SS, Bell GI, Polonsky KS (2001). Molecular mechanisms and clinical pathophysiology of maturity-onset diabetes of the young.. N Engl J Med.

[2] Raeder H, Johansson S, Holm PI, Haldorsen IS, Mas E (2006). Mutations in the CEL VNTR cause a syndrome of diabetes and pancreatic exocrine dysfunction.. Nat Genet..

[3] Weedon MN, Frayling TM (2007). Insights on pathogenesis of type 2 diabetes from MODY genetics.. Curr Diab Rep.

[4] Iynedjian PB (1993). Mammalian glucokinase and its gene.. Biochem J.

[5] Gloyn AL (2003). Glucokinase (GCK) mutations in hyper- and hypoglycemia: maturity-onset diabetes of the young, permanent neonatal diabetes, and hyperinsulinemia of infancy.. Hum Mutat.

[6] Pinterova D, Ek J, Kolostova K, Pruhova S, Novota P (2007). Six novel mutations in the GCK gene in MODY patients.. Clin Genet.

[7] Kamata K, Mitsuya M, Nishimura T, Eiki J, Nagata Y (2004). Structural basis for allosteric regulation of the monomeric allosteric enzyme human glucokinase.. Structure.

[8] Aleshin AE, Zeng C, Bartunik HD, Fromm HJ, Honzatko RB (1998). Regulation of hexokinase I: crystal structure of recombinant human brain hexokinase complexed with glucose and phosphate.. J Mol Biol.

[9] Garcia-Herrero CM, Galàn M, Vincent O, Flàndez B, Gargallo M (2007). Functional analysis of human glucokinase gene mutations causing GCK MODY: exploring the regulatory mechanisms of glucokinase activity.. Diabetologia.

[10] Centre for Disease Control normative curves.. http://www.cdc.gov.

[11] Kuczmarski RJ, Ogden CL, Guo SS, Grummer-Strawn LM, Flegal KM (2002). 2000 CDC Growth Charts for the United States: methods and development.. Vital Health Stat 11..

[12] Shield JP, Temple IK, Sabin M, Mackay, Robinson DO (2004). An assessment of pancreatic endocrine function and insulin sensitivity in patients with transient neonatal diabetes in remission.. Arch Dis Child Fetal Neonatal Ed.

[13] Stoffel M, Froguel PH, Takeda J, Zouali H, Vionnet N (1992). Human glucokinase gene: isolation, characterization, and identification of two missense mutations linked to early-onset non-insulin-dependent (type 2) diabetes mellitus.. Proc Natl Acad Sci U S A.

[14] Primer3 Input 0.4.0.. http://frodo.wi.mit.edu/.

[15] Protein families database of alignments. Pfam.. http://www.sanger.ac.uk/Software/Pfam/.

[16] National Center for Biotechnology Information. NCBI.. http://www.ncbi.nlm.nih.gov/.

[17] Glaser F, Rosemberg Y, Kessel A, Pupko T, Ben-Tal N (2005). The ConSurf-HSSP database: the mapping of evolutionary conservation among homologs onto PDB structures.. Proteins.

[18] Human Genome Variation Society. HGVS.. http://www.hgvs.org/mutnomen/.

[19] den Dunnen JT, Antonarakis E (2001). Nomenclature for the description of human sequence variations.. Hum Genet.

[20] den Dunnen JT, Paalman MH (2003). Standardizing mutation nomenclature: why bother?. Hum Mutat.

[21] Fiser A, Sali A (2003). Modeller: generation and refinement of homology-based protein structure models.. Methods Enzymol.

[22] Berendsen HJ, van der Spoel D, van Drunen R (1995). GROMACS: “A message-passing parallel molecular dynamics implementation”.. Comp. Phys. Comm.

[23] Fraternali F, Van Gunsteren WF (1996). An efficient mean solvation force model for use in molecular dynamics simulations of proteins in aqueous solution.. J Mol Biol.

[24] Prisco F, Iafusco D, Franzese A, Sulli N, Barbetti F (2000). Mody 2 presenting as neonatal hyperglycaemia: a need to reshape the definition of “neonatal diabetes”?. Diabetologia.

[25] Massa O, Meschi F, Cuesta-Munoz A, Caumo A, Cerutti F (2001). High prevalence of glucokinase mutations in Italian children with MODY. Influence on glucose tolerance, first-phase insulin response, insulin sensitivity and BMI.. Diabetologia.

[26] Mantovani V, Salardi S, Cerreta V, Bastia D, Cenci M (2003). Identification of eight novel glucokinase mutations in Italian children with maturity-onset diabetes of the young.. Hum Mutat.

[27] Toaima D, Näke A, Wendenburg J, Praedicow K, Rohayem J (2005). Identification of novel GCK and HNF1A/TCF1 mutations and polymorphisms in German families with maturity-onset diabetes of the young (MODY).. Hum Mutat.

[28] Burke CV, Buettger CW, Davis EA, McClane SJ, Matschinsky FM (1999). Cell-biological assessment of human glucokinase mutants causing maturity-onset diabetes of the young type 2 (MODY-2) or glucokinase-linked hyperinsulinaemia (GK-HI).. Biochem J.

[29] Ellard S, Thomas K, Edghill EL, Owens M, Ambye L (2007). Partial and whole gene deletion mutations of the GCK and HNF1A genes in maturity-onset diabetes of the young.. Diabetologia.

[30] Veiga-da-Cunha M, Courtois S, Michel A, Gosselain E, Van Schaftingen E (1996). Amino acid conservation in animal glucokinases. Identification of residues implicated in the interaction with the regulatory protein.. J Biol Chem..

[31] Velho G, Blanchè H, Vaxillaire M, Bellannè-Chantelot C, Pardini VC (1997). Identification of 14 new glucokinase mutations and description of the clinical profile of 42 MODY-2 families.. Diabetologia.

[32] Miller SP, Anand GR, Karschnia EJ, Bell GI, LaPorte DC (1999). Characterization of glucokinase mutations associated with maturity-onset diabetes of the young type 2 (MODY-2): different glucokinase defects lead to a common phenotype.. Diabetes.

[33] Hattersley AT, Beards F, Ballantyne E, Appleton M, Harvey R (1998). Mutations in the glucokinase gene of the fetus result in reduced birth weight.. Nat Genet.

[34] Sagen JV, Odili S, Bjorkhaug L, Zelent D, Buettger C (2006). From clinicogenetic studies of maturity-onset diabetes of the young to unraveling complex mechanisms of glucokinase regulation.. Diabetes.

[35] The Human Gene Mutation Database at the Institute of Medical Genetics in Cardiff (HGMD).. http://www.hgmd.cf.ac.uk/ac/index.php.

[36] Berger M, Mönks D, Schmidt H, Krane V, Wanner C (2005). Are glucokinase mutations associated with low triglycerides?. Clin Chem.

[37] Rhodes CJ, White MF (2002). Molecular insight into insulin action and secretion.. Eur J Clin Invest.

